# Long-term effects of contrast media exposure on renal failure progression: a retrospective cohort study

**DOI:** 10.1186/s12882-023-03194-2

**Published:** 2023-05-17

**Authors:** Tomoyuki Takura, Kosaku Nitta, Ken Tsuchiya, Hideki Kawanishi

**Affiliations:** 1 Department of Healthcare Economics and Health Policy, Graduate School of Medicine, The University of Tokyo, 7-3-1, Hongo, Bunkyo, Tokyo, 1138655 Japan; 2 Department of Medicine, Kidney Centre, Tokyo Women’s Medical University, 8-1, Kwadacho, Shinjuku, Tokyo, 1628666 Japan; 3 Department Blood Purification, Kidney Centre, Tokyo Women’s Medical University, 8-1, Kwadacho, Shinjuku, Tokyo, 1628666 Japan; 4 Tsuchiya General Hospital, 3-30, Nakajimacho, Naka-ku, Hiroshima, 7308655 Japan

**Keywords:** Long-term effects, Contrast media, Glomerular filtration, Advanced renal failure, Appropriate use criteria, Big data, Propensity score matching

## Abstract

**Background:**

With the constant need for technique improvement for ensuring correct diagnoses and precise treatment, imaging examinations that use contrast media have become unavoidable and indispensable. However, the long-term effects of contrast media on renal function remain unclear in populations with advanced renal failure. This study aimed to examine the relationship between contrast media exposure and long-term trends in renal function in patients with renal failure.

**Methods:**

This retrospective cohort study included patients with a definitive diagnosis of chronic kidney disease who visited medical institutions in Japan between April 2012 and December 2020. The cohort was divided into contrast agent therapy and non-contrast agent therapy groups. The assessment indices were the number of contrast exposures and renal function decline. Renal function decline was calculated based on observed chronic kidney disease stage trends and glomerular filtration rate correspondence tables sourced from various guidelines. A stratified analysis focusing on changes in renal function while accounting for the acceleration of chronic kidney disease progression was also performed.

**Results:**

After adjusting for patient background with propensity score matching, 333 patients each were included in both groups. The observation period was 5.3 ± 2.1 and 4.9 ± 2.2 years per case in the contrast-enhanced and non-contrast-enhanced groups, respectively. The baseline estimated glomerular filtration rate at the beginning of the observation period was 55.2 ± 17.8 mL/min/1.73 m^2^ in the contrast-enhanced groups (*P* = 0.65). Although only slightly different in both groups, the glomerular filtration rate change was 1.1 ± 3.3 mL/min/1.73 m^2^/year in the contrast agent therapy group and tended to be higher with contrast media exposure. Stratified analysis showed that the annual glomerular filtration rate changes in patients with more contrast media exposures and altered renal function were 7.9 ± 7.1 mL/min/1.73 m^2^/year and 4.7 ± 3.6 mL/min/1.73 m^2^/year in the contrast agent therapy and non-contrast agent therapy groups, respectively (1.69 times, *P* < 0.05).

**Conclusion:**

We were able to identify a clinical trend of successful measures for preventing adverse renal outcomes associated with contrast media exposure. However, increased frequency of contrast media exposure has a long-term effect on renal function in patients with altered it. Appropriate treatment choices related to contrast media may control chronic kidney disease.

**Supplementary Information:**

The online version contains supplementary material available at 10.1186/s12882-023-03194-2.

## Background

With the increasing importance of accurate diagnosis and precise treatment, imaging examinations using contrast media have become unavoidable and indispensable in many disease areas. However, contrast media can induce hypersensitivity symptoms such as erythema and urticaria immediately after injection, resulting in severe shock. Despite years of research, the pathogenesis of contrast-induced nephropathy (CIN) remains poorly understood. The risk of CIN development includes chronic kidney disease (CKD), which occurs in 4–11% of patients with moderate renal impairment and 50% of patients with severe renal impairment [1–4]. Management by rehydration is the primary preventive measure for CIN.

The effects of CIN on renal function are often transient [1, 5]. For example, serum creatinine levels peak at 2–4 days after contrast administration and recover to pre-administration values within 2 weeks. In a short-term post-administration analysis of 90–180 days, contrast media testing did not affect renal function decline in a few studies [6–8]. CIN related to percutaneous coronary intervention does not affect the long-term prognosis (death or initiation of permanent dialysis) of Japanese patients with advanced renal failure [9].

In contrast, renal function decline progresses to the point of requiring haemodialysis in some cases. There are scattered reports that CIN affects renal function decline in a long-term analysis over 2 years after administration [10–12]. With the increasing prevalence of lifestyle-related diseases, there is growing interest in the prevention of CKD progression and risk factors for CIN, including diabetes [13]. Contrast agents, which are taxing on the kidneys, and other risk factors, such as diabetes and the use of non-steroidal anti-inflammatory drugs (NSAIDs), are a cause of concern for long-term CKD progression, mainly because of frequent contrast studies and imaging-based treatments for chronic diseases [10].

As described above, concerns have been raised regarding the long-term prognosis of the effect of contrast media on renal function, but sufficient data have not been accumulated. In particular, the effects of contrast media on CIN development and the long-term prognosis (follow-up period of 5 years or more) of CKD have not been fully investigated in populations with advanced renal failure. Therefore, we examined the relationship between medical intervention with contrast media and long-term trends in renal function in patients with renal failure from the perspective of preventing CKD progression.

The present study aimed to analyse the long-term effects of contrast media exposure in the field of CKD, which is of great social concern due to its high disease and economic burdens, and to compile basic data that will contribute to the study of preventive measures against its progression. The obtained results will indirectly contribute to the development of medical strategies for the application of contrast media testing and study of disease mechanisms, such as CIN.

## Methods

### Research design

This study was a retrospective cohort study (data science) that applied medical big data (*TheBD*: The Tokyo University Health Economy Big Data) for real-world evaluation. The study data were collected from April 2012 to December 2020, and the target population included patients diagnosed with CKD (International Classification of Diseases, 10th edition [ICD-10] code: N18) who visited medical institutions (examination and treatment intervention) in Japan. CKD is defined as ‘renal impairment evident by urinalysis, blood tests, or imaging’; ‘glomerular filtration rate (GFR) less than 60 ml/min/1.73 m^2^’, or both of these findings for at least 3 months. The exclusion criteria were as follows: (1) individuals aged < 20 years who had congenital renal disease; (2) individuals receiving renal replacement therapy; and (3) individuals with cancer (within 3 years prior to observation), except for renal cancer, which was excluded from the sample of patients with cancer. Samples for which the use of iodine contrast media could not be correctly determined were also excluded. We adhered to the STROBE guidelines for this cohort study.

The cohort was divided into contrast agent therapy (CAT, contrast-enhanced treatment) and non-contrast agent therapy (non-CAT, non-contrast-enhanced treatment) groups for laboratory and therapeutic interventions while considering the risk factors for CKD, such as hypertension, diabetes, medications (e.g., analgesics and antihypertensive drugs), age, and sex. Renal function decline was calculated from the correspondence table between the CKD stage (KDIGO 2012: G1–G5) and GFR reference values (CKD Practice Guide 2012: mL/min/1.73 m^2^) [14, 15]. The endpoints were the number of contrast exposures, regardless of contrast route and dose, and renal function derangement. They were analysed in units of annual averages divided by the observation period, accounting for the timing of reciprocal occurrences. The framework was a long-term longitudinal study based on disease characteristics and study objectives. The average analysis period was 5 years, with a minimum observation period of 6 months or longer.

### Study data source

This study used a large database that includes medical service data examined by a specialised public organisation (Social Insurance Medical Fee Payment Fund), in accordance with the format stipulated by the Ministry of Health, Labor and Welfare (MHLW Notification: Vol. 0831 No. 1). We selected medical economic big data (*TheBD*; S1 Table), [16, 17] which included medical service bills in Japan between April 2012 and March 2021, representing the coverage for 7 million insured patients.

The present study was conducted in accordance with the principles embodied in the Declaration of Helsinki. This study received comprehensive approval in March 2019 from the Institutional Review Board of the University of Tokyo Hospital (screening no: 2018167NI, approval date: March 26, 2019). Because we used database records (anonymized) for analysis, the requirement for the acquisition of informed consent from patients was waived at the above Institutional Review Board (opt-out format).

The largest proportion of the sample was from the year 2016 (22.1%). Medical information accounted for 6.18 million results while dispensing information accounted for 6.20 million results (including duplications). The patient-based hospitalisation rate was 13.5% (including duplications), and the average percentage of male patients for all years was 46.8%. This database is updated every 6 months. All data on disease name, testing, medication, surgery, and any other medical interventions with dates of initiation and related costs are linked in chronological order using unique IDs for each patient. During each biannual update, the transfer of data for insured persons is managed, and adjustments are made according to the allocation of medical facilities. *TheBD* has been used in several studies evaluating the economic aspects of medical interventions (S2 Table).

### Selection conditions for research subjects

For cases that met the abovementioned selection criteria but did not meet the exclusion criteria, we defined the observation period to be at least 6 months from the date of the first diagnosis of CKD stage. Injuries were identified by the ICD-10 code (N18) and CKD stage (injury name).

The analysis period was from the date when the CKD stage injury was first identified (index day) or the date of data generation, whichever was earlier, to the date when renal replacement therapy was initiated. We observed hemodialysis, peritoneal dialysis, and renal transplantation interventions as renal replacement therapies. For patients who did not receive renal replacement therapy, the analysis period was up to the date of the last identifiable CKD stage injury (end date).

**Fig. 1:**
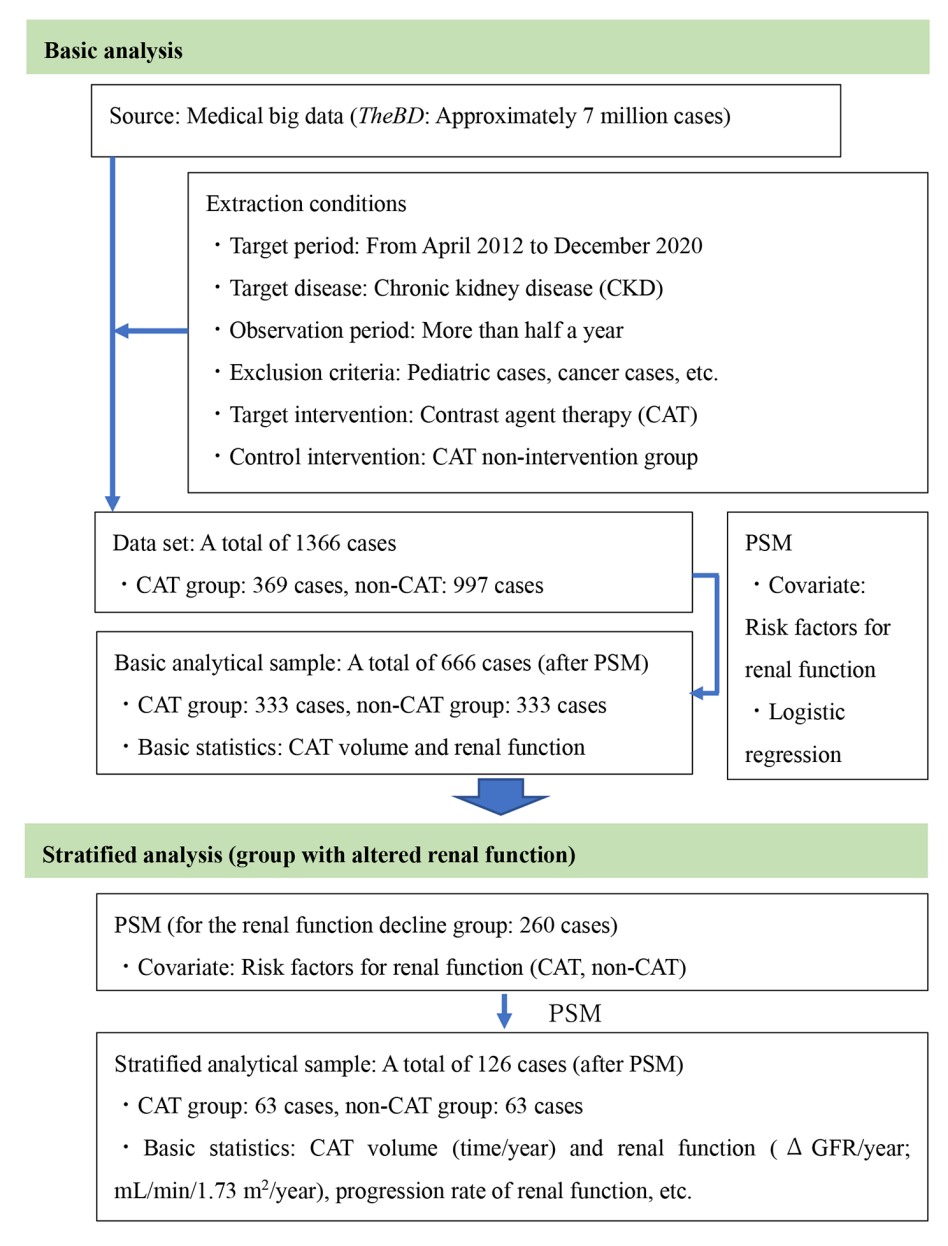
Structure of this study: basic flow of data set and main analysis conditions PSM, propensity score matching

Data extraction was performed on the six components (conditions) listed in the ‘Extraction conditions’ table shown in Fig. 1. We first selected patients (15095 cases) with renal failure that met the selection criteria from the medical big data (Fig. 1). We then excluded those that met the exclusion criteria, for a final sample of 1366 cases. We classified this population into CAT and non-CAT groups according to contrast exposure. The patient background in both groups was adjusted for major risk factors for renal function by applying the propensity score matching (PSM) method.

Thus, this study included 333 patients in the CAT and non-CAT groups, totalling to 666 patients. In this study, most CATs were imaging tests. We also performed a stratified analysis focusing on the group with altered renal function (260 patients; 39.0%), while taking the acceleration of CKD progression into account. For the stratified analysis (260 patients), the decline in renal function group was selected from the basic analysis cohort (666 patients). After processing PSM, the analysis included 63 patients in each group, totalling 126 patients.

We calculated the cut-off value of renal function decline for CAT from the receiver operating characteristic (ROC) curve in the stratification analysis.

### Evaluable interventions and precautionary conditions

The interventions evaluated in this study were practices involving iodine contrast media. All iodine contrast media approved in Japan were included in the evaluation.

Iodine contrast media are classified into two types: water-soluble and oil-based. Water-soluble contrast media are used in patients with normal renal function. Approximately 90% is excreted as urine from the kidneys at approximately 6 h after injection. In contrast, oil-based iodine contrast media can remain in the body for a long period. These two types of contrast media are used separately according to the purpose of the examination. Water-soluble contrast media are selected in most cases. Water-soluble contrast media can be further classified into ionic and non-ionic contrast media. Compared with ionic contrast media, non-ionic contrast media have lower osmotic pressure and are more similar to blood, which alleviates the symptoms of adverse reactions, such as the burning sensation experienced during injection. In Japan, non-ionic contrast media are most commonly used in angiography.

During the examination of contrast exposure and its impact on renal function, the amount of contrast media injected into the body is of importance. However, the data source for this study is based on information from medical claims, which provides information on product consumption but not on internal infusion volume. In addition, information on weight and detailed imaging type of the subject cases is essential for determining the internal infusion volume. However, due to the large uncertainty, there were limitations regarding the organisation of this information.

The rehydration (saline) volume was categorised by drug category (code 331), and the cumulative volume (in mL/year) within the observation period was calculated. Consistency was also maintained for rehydration on a yearly basis in relation to the analysis of time-series changes in renal function. This volume was calculated from the volume set for each drug name, as there was variation in the volume or number of times that it was used, depending on the case or prescription. Furthermore, if the frequency was two or more times, it was further corrected by considering the frequency in addition to the calculated amount used. The figures for this calculation are the product consumption, not the injectable volume.

### Main analysis methods and assessment of renal function

The Mann–Whitney U test was used to evaluate the effect of iodine contrast media use on renal function, and the chi-square test was used along with the previously described test to compare patient background. The significance level was set at 5%. We calculated Pearson’s correlation coefficient to assess the correlation between the number of contrast media used and the decline in renal function. Multivariate analysis of risk factors for renal function was performed using logistic regression analysis. Statistical analysis was performed using SPSS version 26.0 (IBM Corp., Armonk, New York). The figures in the tables are presented as means and standard deviations, whereas the figures are presented as standard errors.

PSM was used to minimise bias related to patient background. Therefore, the forced entry method was chosen for the multivariate logistic regression model. The independent variables (covariates) were age, sex, hypertension/diabetes, use of NSAIDs, antimicrobial agents, antineoplastic agents, and others. Propensity scores for each case were calculated, and a 1:1 matching method was applied to align the sample numbers and confirm the data distribution and balance in both groups. Among the independent variables (covariates), age was defined as the date of CKD stage; hypertension was diagnosed within the observation period; and the use of NSAIDs, antimicrobial agents, and antineoplastics within the observation period was defined as the scope of analysis.

The GFR in each case was converted from the GFR category correspondence table of CKD severity classification based on the observed CKD stage in ICD-10 codes in this study. Furthermore, we estimated the decline in renal function associated with changes in CKD stage from the CKD stages and GFR levels in previous studies [18–21]. The underlying data used for the conversion are shown in Table 1. The annual change in estimated GFR was calculated as follows: [ΔGFR: estimated GFR (index day) - estimated GFR (end date)] / [duration: observation period]. When some information regarding the CKD stage was missing, estimates were made based on pre- and post-stage data.

**Table 1:** CKD stage to GFR conversion

Item	Conversion of GFR from CKD stage						
CKD stage	G1	G2	G3a	G3b	G4	G5	G5D^a^
GFR criteria (mL/min/1.73 m^2^)	100	89	59	44	29	14	5
Amount of decline in renal function associated with changes in CKD stage (ΔGFR)	11	30	15	15	15	9	―

^a^ Renal replacement therapy, haemodialysis, peritoneal dialysis, renal transplantation

## Results

### Basic analysis of the whole population

**Fig. 2:**
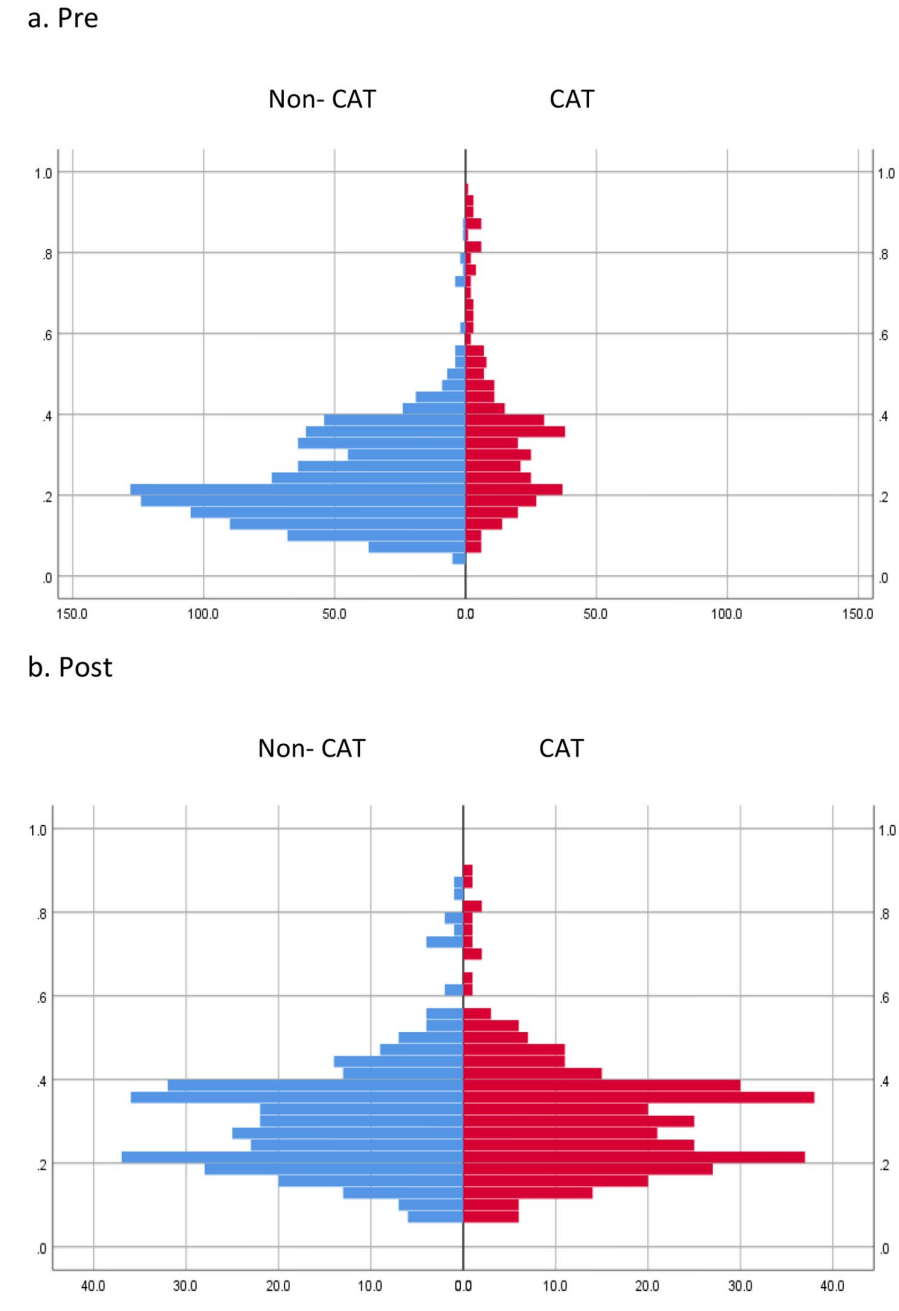
Pre- and post-matching covariates for both groups by PSM (in the basic analysis)

The analysis population comprised 1366 patients (CAT group: *n* = 369, non-CAT group: *n* = 997). After adjusting for patient background with PSM, 333 patients (age: 55.6 ± 9.0, male percentage: 70.5%) were included in the CAT group, whereas 333 patients (age: 56.0 ± 8.6, male percentage: 73.0%) were included in the non-CAT group (*P* = 0.48 and 0.55 for age and sex, respectively) (Table 2). Figure 2 shows the covariates before and after matching by PSM in both groups. The duration of observation was 5.3 ± 2.1 and 4.9 ± 2.2 years per case in the CAT and non-CAT groups, respectively (*P* < 0.05). The mean number of contrast exposures was 2.4 ± 2.4 per case (mean per year: 0.45 per case). The volume of contrast media was 46.2 ± 54.6 mL/procedure/year. The baseline estimated GFR at the beginning of observation was 55.2 ± 17.8 mL/min/1.73 m^2^ and 53.3 ± 15.2 mL/min/1.73 m^2^ in the CAT and non-CAT groups, respectively (*P* = 0.65).

**Table 2:** Basic analysis of patient background and various interventions

	Parameter	CAT group			Non-CAT group			*P*-value
Case								
	Sample (*n*)	333			333			
	Age (years)	55.6	±	8.6	56.0	±	8.6	0.48
	Sex: male (%)	70.9			73.0			0.55
	Observation period (years)	5.3	±	2.1	4.9	±	2.2	0.04
	Baseline estimated GFR (mL/min/1.73 m^2^)	55.2	±	17.8	53.3	±	15.2	0.65
Main disease								
	Diabetic nephropathy (%)	8.4			6.3			0.30
	Glomerular nephritis (%)	15.9			18.6			0.36
	Nephrosclerosis (%)	4.8			4.8			1.00
	Hypertension (%)	84.1			86.8			0.32
	Diabetes (%)	39.0			36.9			0.58
Contrast agent								
	Number of contrast agent exposures (time/year)	2.4	±	2.4				―
	Amount of contrast agent (mL/year)	46.1	±	54.6				―
	Amount of rehydration (mL/year)	1689.5	±	3399.7	289.0	±	900.9	< 0.01
Main medicine								
	Hyperkalaemia drug (%)	12.9			14.7			0.50
	Phosphate binder (%)	6.6			8.1			0.46
	Erythropoiesis-stimulating agent (%)	15.0			18.0			0.30
	Spherical carbon adsorbent (AST-120) (%)	6.6			11.1			0.04
	Antihypertensive (%)	75.7			76.9			0.72
	Diuretic (%)	37.5			28.8			0.02
	Diabetes drug (%)	31.2			29.1			0.56
	Non-steroidal anti-inflammatory drug (%)	62.8			60.4			0.52
	Antibacterial drug (%): Aminoglycosides, Glycopeptides, Others	9.6			7.2			0.26
Main surgery								
	Revascularisation (%)	2.4			0.0			0.05
	Cardiovascular implantable electronic device, others (%)	1.2			0.6			0.77
	Heart valve replacement, valvuloplasty (%)	0.3			0.0			0.85
Main prevention								
	Lifestyle disease prevention (education, guidance) (%)	3.6			1.8			0.69
CKD stage (final in the observation period: end day)								
	G1 (%)	2.7			2.7			1.00
	G2 (%)	12.6			14.4			0.50
	G3a (%)	40.8			39.3			0.69
	G3b (%)	16.5			12.3			0.12
	G4 (%)	10.2			11.4			0.62
	G5 (%)	4.2			5.7			0.37
	G5D (%)	12.9			14.1			0.65

The underlying cause of renal failure was glomerulonephritis in 15.9% and 18.6% of the patients in the CAT and non-CAT groups, respectively (*P* = 0.36). Diabetic nephropathy was observed in 8.4% and 6.3% of the patients in the CAT and non-CAT groups, respectively (*P* = 0.30). Concomitant hypertension was observed in 84.1% and 86.8% of the patients in the CAT and non-CAT groups, respectively (*P* = 0.32). The main drugs were NSAIDs in 62.8% and 60.4% of the patients in the CAT and non-CAT groups, respectively (*P* = 0.52); antihypertensives in 75.7% and 76.9% of the patients in the CAT and non-CAT groups, respectively (*P* = 0.72); and antidiabetic drugs in 31.2% and 29.1% of the patients in the CAT and non-CAT groups, respectively (*P* = 0.56).

Disease prevention interventions were performed in 1.8% and 1.2% of the patients in the CAT and non-CAT groups (*P* = 0.52), respectively, for lifestyle guidance (comprehensive management including education). The rehydration volume was 1689 ± 3399 mL/year and 289 ± 900 mL/year in the CAT and non-CAT groups, respectively (*P* < 0.01).

The final CKD stage during the observation period was 12.9% and 14.1% (*P* = 0.65) in renal replacement therapy (G5D); 4.2% and 5.7% (*P* = 0.37) in G5; 10.2% and 11.4% (*P* = 0.62) in G4; 16.5% and 12.3% in G3b; and 40.8% and 39.3% (*P* = 0.69) in G3a in the CAT and non-CAT groups, respectively.

**Fig. 3:**
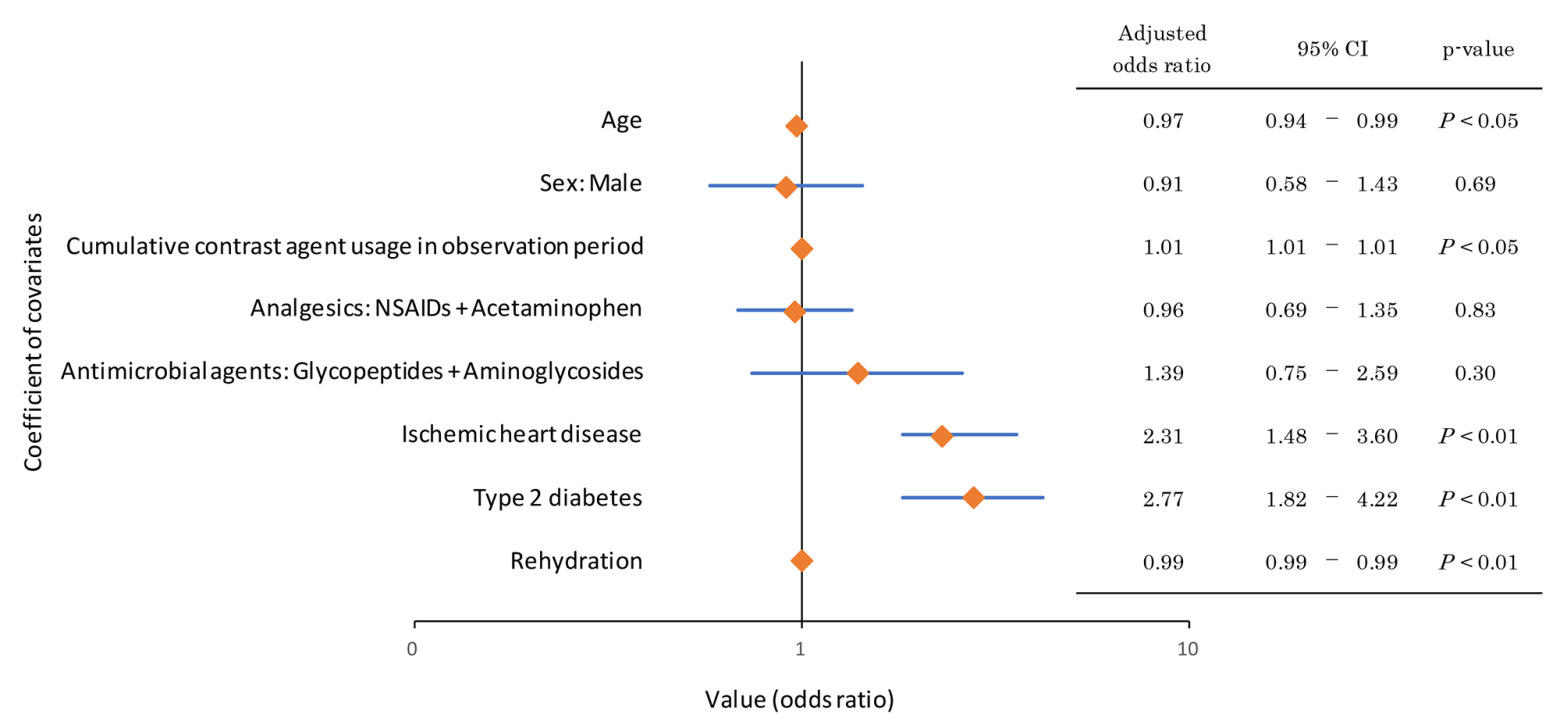
Odds ratios of each covariate for renal function decline (logistic regression, *n* = 666) NSAID, non-steroidal anti-inflammatory drug. OR, odds ratio Note: Age: year, Sex: binary (Male), Cumulative contrast agent usage: mL/year, Analgesics: binary, Antimicrobial agents: binary, Ischemic heart disease: binary, Type 2 diabetes: binary, Rehydration: mL/year

**Fig. 4:**
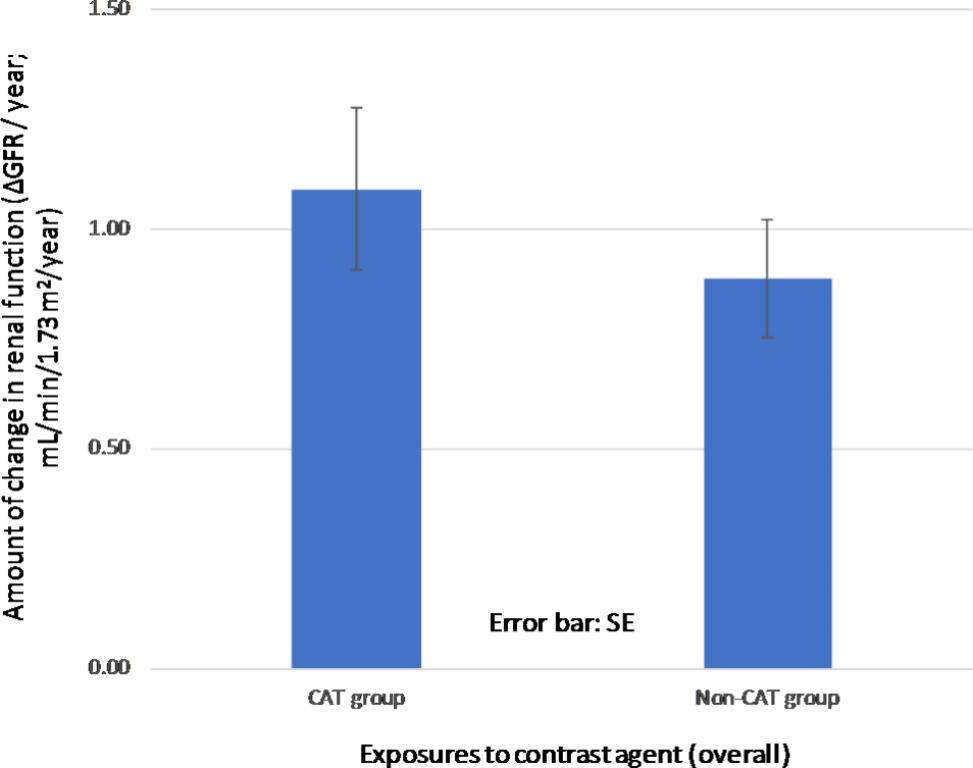
Annual GFR decline by contrast exposure (basic analysis, after PSM, *n* = 666) SE: standard error

Logistic regression analysis of this population, with renal function decline (presence or absence of declined renal function during the observation period) as the objective variable, resulted in an odds ratio of 1.01 (95% confidence interval: 1.01–1.01, *P* < 0.05) for the cumulative contrast media usage in the observation period (Fig. 3). Thus, the annual GFR change was 1.1 ± 3.3 mL/min/1.73 m^2^/year and 0.9 ± 2.5 mL/min/1.73 m^2^/year in the CAT and non-CAT groups (*P* = 0.91), respectively (Fig. 4 ). It was implied that an increase in contrast media exposure might have an increased effect on renal function decline. For example, the population with more than 0.3 contrast media exposures per year had a decline in renal function to 1.3 ± 4.2 mL/min/1.73 m^2^/year in the CAT group.

### Stratified analysis of the group with altered renal function

**Fig. 5:**
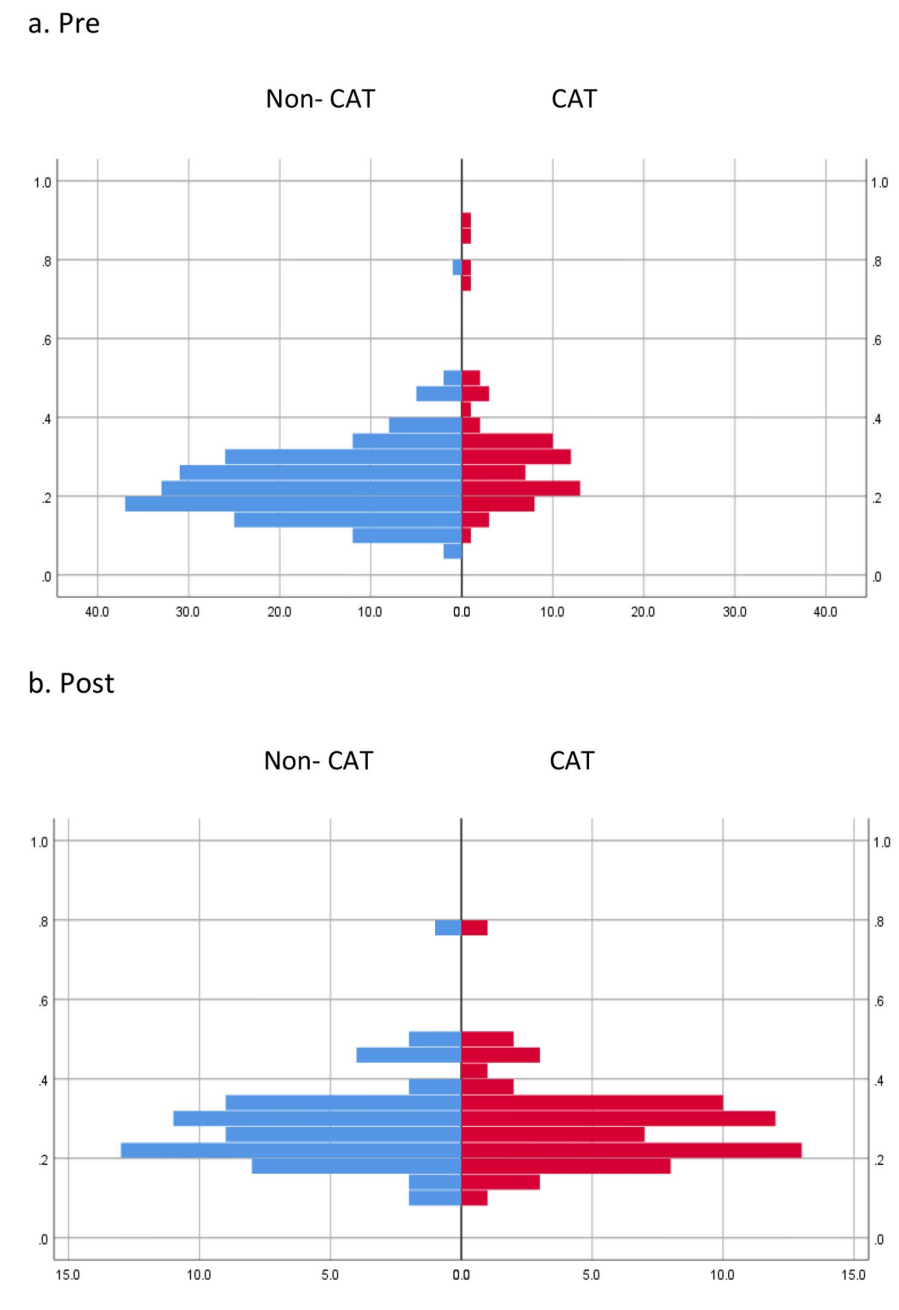
Pre- and post-matching covariates for both groups by PSM (stratified analysis)

In the renal function decline group, the target population for stratified analysis, 63 patients (age: 54.4 ± 9.0, male ratio: 71.4%) were in the CAT group, and 63 patients (age: 54.5 ± 7.9, male ratio: 77.8%) were in the non-CAT group (*P* = 0.93 and 0.41 for age and sex, respectively) after adjusting for patient background with PSM (Table 3). Figure 5 presents the pre- and post-matching covariates for both groups by PSM. The observation period was 5.1 ± 2.0 years and 4.6 ± 1.8 years per case in the CAT and non-CAT groups (*P* = 0.16), respectively. The mean number of contrast exposures was 1.9 ± 1.7 per case (mean per year: 0.37 per case). The volume of contrast media was 40.1 ± 54.1 mL/procedure/year.

**Table 3:** Stratified analysis of patient background and various interventions (population with renal function displacement)

	Parameter	CAT group			Non-CAT group			*P*-value
Case								
	Sample (*n*)	63			63			
	Age (years)	54.4	±	9.0	54.5	±	7.9	0.93
	Sex: male (%)	71.4			77.8			0.41
	Observation period (years)	5.1	±	2.0	4.6	±	1.8	0.16
	Baseline estimated GFR (mL/min/1.73 m^2^)	42.1	±	46.6	31.5	±	41.4	0.22
Disease								
	Diabetic nephropathy (%)	27.0			20.6			0.40
	Glomerular nephritis (%)	22.2			12.7			0.16
	Nephrosclerosis (%)	1.6			6.3			0.37
	Hypertension (%)	98.4			100.0			1.00
	Diabetes (%)	60.3			61.9			0.86
Contrast agent								
	Number of contrast agent exposures (time/year)	1.9	±	1.7				―
	Amount of contrast agent (mL/year)	40.1	±	54.2				―
	Amount of rehydration (mL/year)	3983.4	±	5635.8	969.1	±	1264.9	< 0.01
Main medicine								
	Hyperkalaemia drug (%)	42.9			49.2			0.48
	Phosphate binder (%)	30.2			39.7			0.26
	Erythropoiesis-stimulating agent (%)	63.5			69.8			0.45
	Spherical carbon adsorbent (AST-120) (%)	27.0			36.5			0.25
	Antihypertensive (%)	88.9			95.2			0.19
	Diuretic (%)	69.8			58.7			0.19
	Diabetes drug (%)	58.7			55.6			0.72
	Non-steroidal anti-inflammatory drug (%)	57.1			58.7			0.86
	Antibacterial drug (%): Aminoglycosides, Glycopeptides, Others	15.9			11.1			0.43
	Anticancer drug (%)	1.6			1.6			1.00
Main surgery								
	Revascularisation (%)	12.1			0.0			< 0.01
	Cardiovascular implantable electronic device, others (%)	6.9			0.9			< 0.01
	Heart valve replacement, valvuloplasty (%)	2.2			0.0			0.06
Main prevention								
	Lifestyle disease prevention (education, guidance) (%)	6.3			3.2			0.68

*Note*: Most CATs in this study were imaging tests. Therefore, descriptions in this table focused on therapeutic interventions

The baseline estimated GFR at the beginning of observation was 42.1 ± 46.6 mL/min/1.73 m^2^ and 31.5 ± 41.4 mL/min/1.73 m^2^ in the CAT and non-CAT groups (*P* = 0.22), respectively. The GFR level of the non-CAT group was lower than that of the CAT group in baseline.

The underlying cause of renal failure was diabetic nephropathy in 27.0% and 20.0% of the patients in the CAT and non-CAT groups (*P* = 0.40), respectively, and glomerulonephritis in 22.2% and 12.7% of the patients in the CAT and non-CAT groups (*P* = 0.16), respectively. Concomitant hypertension was observed in 98.4% and 100.0% of the patients in the CAT and non-CAT groups, respectively (*P* = 1.00). The main drugs were NSAIDs in 57.1% and 58.7% (*P* = 0.86) and antihypertensive drugs in 88.9% and 95.2% (*P* = 0.19) of the patients in the CAT and non-CAT groups, respectively. Other major therapeutic interventions included revascularisation (e.g., percutaneous coronary intervention) (12.4%), cardiovascular implantable electronic device (6.9%), and valve replacement/valvuloplasty (2.2%).

Disease prevention interventions accounted for 6.3% and 3.2% in the CAT and non-CAT groups (*P* = 0.68), respectively, for lifestyle guidance (mostly diabetes dialysis prevention guidance and management). The rehydration volume was 3983 ± 5635 mL/year and 969 ± 1264 mL/year in the CAT and non-CAT groups, respectively (*P* < 0.01).

**Fig. 6:**
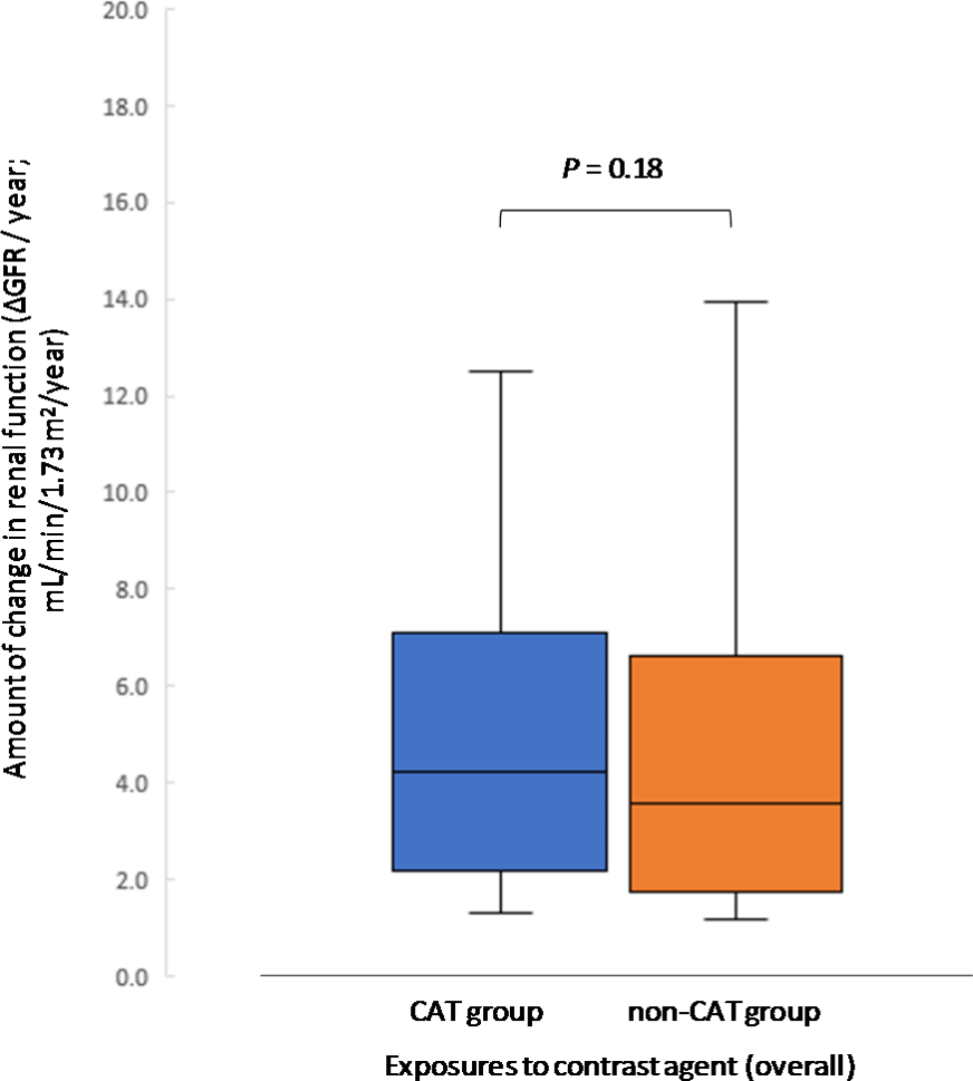
Comparison of annual GFR change by contrast exposure (stratified analysis, after PSM, *n* = 126)

**Fig. 7:**
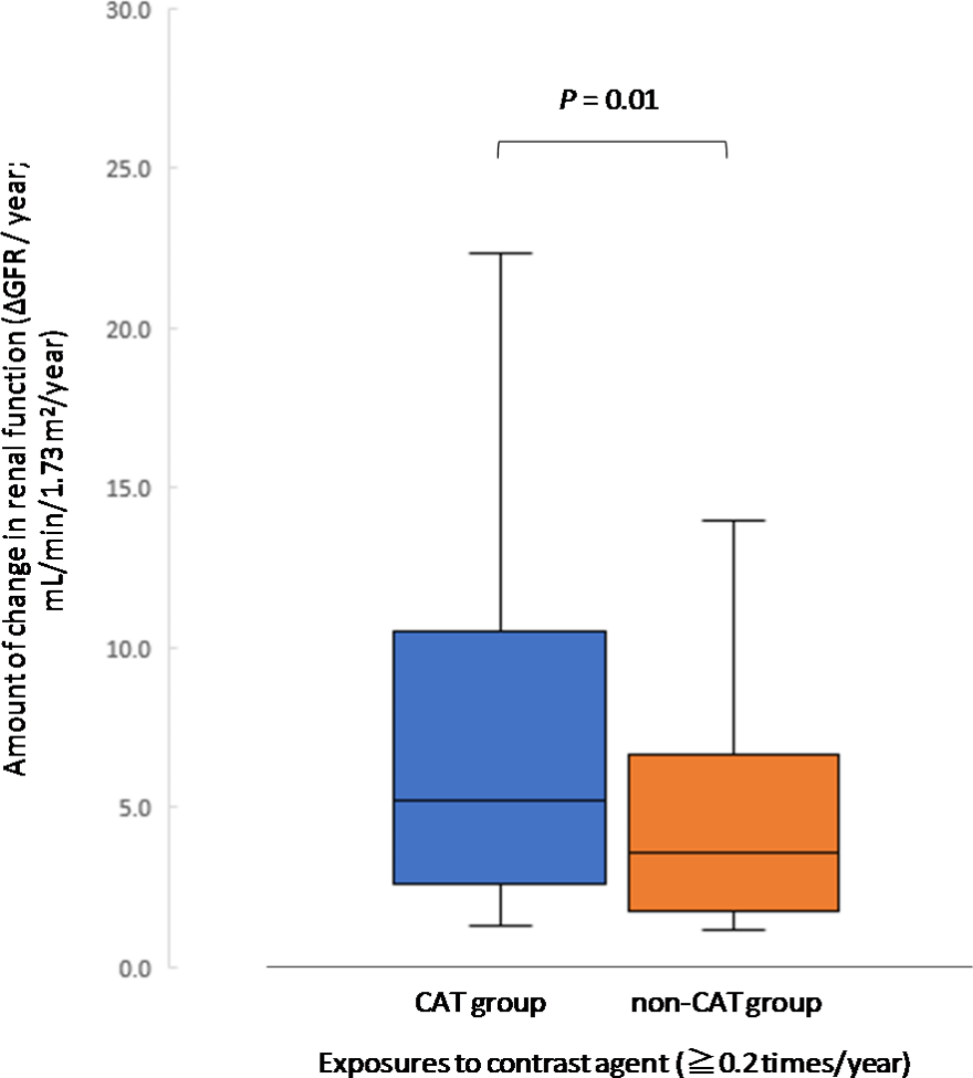
Comparison of annual GFR change (stratified analysis, population with > 0.2 contrast exposures per year)

In the renal function decline group, the annual GFR change was 5.8 ± 5.6 mL/min/1.73 m^2^/year and 4.7 ± 3.6 mL/min/1.73 m^2^/year in the CAT and non-CAT group, respectively (*P* = 0.18) (Fig. 6). Building on the supporting results of the basic analysis, we also attempted an analysis in a population with high exposure to contrast media. The cut-off value for renal function decline for CAT was in the 0.2 range (times/year, area under the curve: 0.74, 95% CI: 0.62–0.86, *P* < 0.01). We set a cut-off value of 0.2 times/year as a conservative estimate. Although limited by the small sample size (n = 32), in the population with more than 0.2 contrast exposures per year, the CAT group accounted for 7.9 ± 7.1 mL/min/1.73 m^2^/year (*P* < 0.05) (Fig. 7).

**Fig. 8:**
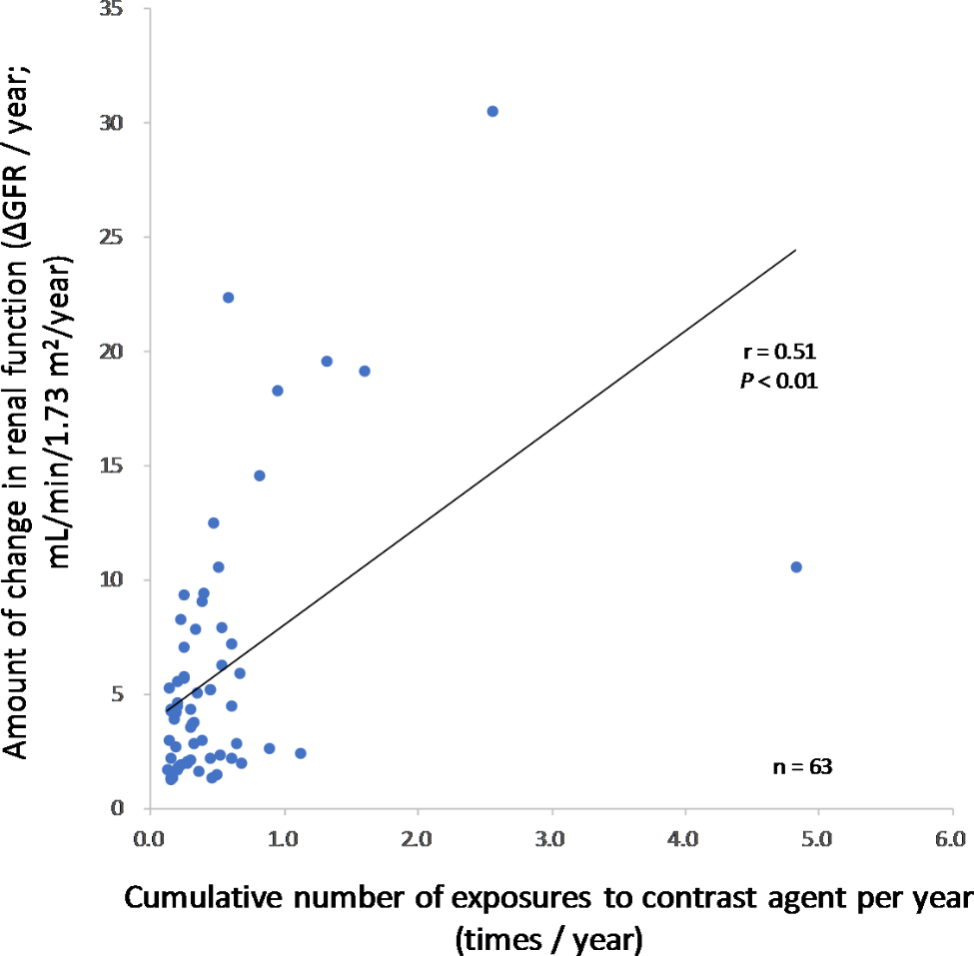
Correlation analysis between the yearly number of contrast exposures and annual GFR change (stratified analysis)

Correlation analysis showed that the number of contrast media exposures per year was statistically significantly positively correlated with the annual GFR change (*r* = 0.51, *P* < 0.01) (Fig. 8).

## Discussion

Notwithstanding the limitations surrounding the collection of information on patient background, test results, some aspects of the clinical picture, and statistics, this study, oriented towards real-world considerations in the Japanese health care system, clarified the actual long-term effects of contrast media exposure on renal function. Thus, we were able to identify a clinical trend of successful measures for preventing renal outcomes associated with contrast media exposure. We subsequently found that contrast exposure under certain conditions is a potential long-term influencing factor on renal function.

Although study design limitations require careful interpretation, the results of longitudinal observations over approximately 5 years have implied that, as with other risk factors, the number of contrast exposures has a long-term effect on renal function. The results of PSM-adjusted stratified analysis (G3b-centred group with altered renal function) showed that the decrease in GFR in the CAT group was 1.69-fold greater than that in the non-CAT group in the population with high annual contrast exposure. Therefore, it was inferred that the strategy of selecting appropriate contrast-enhanced medical treatment is important as a part of CKD measures in moderate CKD and beyond.

The population in this study consisted of CKD patients at high risk of CIN, particularly those in CKD stage G3 and beyond. For urgent diagnosis and treatment in this population, the choice is generally based on a balance of clinical risks and benefits while protecting the renal function [6]. In the present study, we could not examine the amount of contrast media injected into the body and the type of intervention and route of administration in detail. Consequently, we did not discuss in detail the causal relationship between the amount of contrast media exposure and the amount of decline in renal function. Therefore, the results of this study are considered insufficient to contribute to the discussion of clinical decisions as described above. In the future, long-term clinical studies with multivariate analysis of relevant factors (serum creatinine, serum albumin, body mass index, saline, etc.) are desirable [9, 22].

CKD is a concept regardless of the underlying disease, and treatment principles, such as blood pressure, blood glucose control, and salt reduction guidance, remain common [23]. In this study, while comparing the CAT and non-CAT groups, the main risk factors for CKD were adjusted for both groups using PSM. This adjustment excluded lifestyle guidance (including nutritional management, exercise therapy, etc.), which is expected to prevent renal failure progression; however, as a result, there was no significant difference between the two groups. In contrast, factors related to daily life could not be considered due to a lack of data. Further, the proportion of patients in the non-CAT group was significantly higher than that in the CAT group due to insufficient adjustment for AST-120, which inhibits renal failure progression in the two groups. These uncertainties must be considered when interpreting the results of this study. In the future, the new and old prophylactic effects on contrast media exposure must be extensively examined, considering the trend of N-acetylcysteine and others [24, 25].

Although limited by the small sample size, in the analysis of the present study, a more pronounced trend was observed in the groups with greater renal failure progression or a higher number of exposures to contrast media. This trend was also significant in the correlation analysis between the frequency of exposure and renal function decline. In contrast, the frequency of exposure in the group with greater renal function derangement was lower than that in the population as a whole. Considering the relationship between the baseline renal function and renal outcome of contrast exposure, as well as previous reports referring to the relationship between CKD and CIN, the aforementioned trends reiterate the importance of patient background (severity and comorbidity) in the analysis, as well as the clinical significance for the prevention of CKD progression and the use of contrast media [4, 26–28]. The results of this study were interpreted in the same way as the results of the previous study. Therefore, consideration should be given to the possibility that the CAT group has an inherent bias in terms of the severity of renal failure and the need for therapeutic intervention, and a multifaceted discussion of the level of acceleration of CKD progression is also desirable.

Although not many reports have focused on the acceleration of CKD progression, small doses of iodine contrast media (median: 12 mL/dose) for patients with advanced renal failure after stage G4 did not affect the renal outcome; however, an average monthly reduction of 0.14 mL/min/1.73 m^2^ was observed in the eGFR [7]. In the present study, in the overall population, including patients with low exposure, there was no significant difference between the two groups, with an average monthly decrease in progression acceleration of 0.07 mL/min/1.73 m^2^ and 0.09 mL/min/1.73 m^2^ in the non-CAT and CAT groups, respectively. In contrast, in the population with higher exposure frequency, the progression acceleration in the CAT group increased to 0.11 mL/min/1.73 m^2^. A significant effect of contrast exposure (0.48 mL/min/1.73 m^2^/month) was observed in the advanced renal failure group (stage G3b or later) with a higher frequency of contrast exposure. Thus, the progression acceleration of CKD is more pronounced in patients with higher contrast exposure [2–4].

For example, a common renal injury caused by NSAIDs is ischemic nephropathy due to cyclooxygenase inhibition, presenting with acute renal injury. In addition to ischemic nephropathy, acute interstitial nephritis, nephrotic syndrome with interstitial nephritis, and acute tubular necrosis may develop. Previous studies have shown conflicting results, but the overall long-term administration has a significant effect on renal function [29–31]. As shown in the examples above, it is necessary to clearly define factors such as long-term administration and one-time administration; however, it is difficult to comprehensively deal with the effects of administration intervals and doses for each drug type. Our study did not adjust for patient characteristics to account for the duration of other medications. This point represents one of the limitations of this study.

Although this study examined iodine contrast media, other interventions that may have long-term effects on renal function warrant further investigation. For example, fluorescent angiography using sodium fluorescein dye is generally considered safe for patients with renal disease, although there have been scattered reports on the risk of inducing CIN [32]. It is expected that some of findings of this study will be utilised in such exploratory discussions in the CKD field. The data science research that can be developed relatively efficiently is highly significant in organising the basic data to contribute to the initial hypothesis testing and study design. The results of this study will be useful for further discussion in the future, as disease control measures are important from clinical and economic perspectives.

## Conclusion

The present study was a longitudinal observation of the frequency of contrast media exposure and renal function decline over 5 years. As a result of this study, it was clarified that preventive measures against the decline in renal function associated with contrast media exposure are clinically successful over the long term. The results also imply that the number of contrast administration interventions has a long-term effect on the renal function, similar to other risk factors. However, careful interpretation is required due to analytical limitations. Nonetheless, it can be inferred that the promotion of appropriate practice choices related to contrast media is even more important as part of CKD control measures in moderate CKD and beyond.

## Electronic supplementary material

Below is the link to the electronic supplementary material.

Supplementary Material 1

## Data Availability

The datasets used and/or analysed during the current study are available from the corresponding author on reasonable request.

## Abbreviations

CIN: Contrast-induced nephropathy
CKD: Chronic kidney disease
NSAIDs: Non-steroidal anti-inflammatory drugs
ICD-10: International Classification of Diseases, 10th edition
CAT: Contrast agent therapy
non-CAT: Non-contrast agent therapy
GFR: Glomerular filtration rate
MHLW: Ministry of Health, Labor and Welfare
TheBD: The Tokyo University Health Economy Big Data
PSM: Propensity score matching
ROC: Receiver operating characteristic
