# Supplementary material for: Long-term effects of contrast media exposure on renal failure progression: a retrospective cohort study
Source: BMC Nephrol. 2023 May 17;24:135. doi: 10.1186/s12882-023-03194-2 (PMC10189938; doi:10.1186/s12882-023-03194-2)
Supplement: Supplementary file 1 — Supplementary Material 1 [file 12882_2023_3194_MOESM1_ESM.docx]

**Supplementary Materials**

**Supporting information captions:**

**S1 Table.** Database overview (*TheBD*)

**S2 Table.** List of research achievements applying *TheBD* (limited to recent studies [2021 and later])

**S1 Table.** Database overview (*TheBD*)

| **Male/female ratio (overall)** | |  |
| --- | --- | --- |
| **Sex** | **Total number of patients** | **Ratio** |
| **Total** | **7071** | **100.0%** |
| Male | 3337 | 47.2% |
| Female | 3733 | 52.8% |
| **Service type (medical, dentistry, dispensing)** | | |
| **Receipt type** | **Total number of patients** | **Ratio** |
| **Total** | **7071** | **100.0%** |
| Medical | 6816 | 96.4% |
| Dentistry | 4221 | 59.7% |
| Dispensing | 6201 | 87.7% |
| **Inpatient/outpatient ratio (medical)** | |  |
| **Inpatient/outpatient** | **Total number of patients** | **Ratio** |
| **Total** | **7071** | **100.0%** |
| Inpatient | 1046 | 14.8% |
| Outpatient | 7064 | 99.9% |

1. Unit: Notation is “a total of approximately 1000 people” (patient sample).
2. Conditions: In aggregation, the results were narrowed down under the following conditions.

　・An aggregation period of 2012–2019

　・Excluding single samples not used for longitudinal studies

　・Excluding single dental samples

**S2 Table.** List of research achievements applying *TheBD* (limited to recent studies [2021 and later])

*TheBD* has been used in several studies that have evaluated the economic aspects of medical interventions.

1. Takura T, Horiuchi S. Cost-effectiveness analysis of infliximab for the treatment of Kawasaki disease refractory to the initial treatment: a retrospective cohort study. J Cardiol. 2022;80:172–8. https://doi.org/10.1016/j.jjcc.2022.03.005.

2. Ikeuchi K, Okushin K, Saito M, Adachi E, Tsutsumi T, Takura T, et al. Prevalence of HIV infection among non-elderly individuals with hepatitis C in Japan: a population-based cohort study using a health insurance claim data. BMC Infect Dis. 2022;22:167. https://doi.org/10.1186/s12879-022-07152-5.

3. Takura T, Goto K, Honda A. Development of a predictive model for integrated medical and long-term care resource consumption based on health behaviour: application of healthcare big data of patients with circulatory diseases. BMC Medicine. 2021;19:15. https://doi.org/10.1186/s12916-020-01874-6.

4. Takura T, Yokoi H, Tanaka N, Matsumoto N, Yoshida E, Nakata T, et al. Health economics-based verification of functional myocardial ischemia evaluation of stable coronary artery disease in Japan: A long-term longitudinal study using propensity score matching. J Nucl Cardiol. 2022;29:1356–69. https://doi.org/10.1007/s12350-020-02502-9.

5. Shiina T, Goto-Hirano K, Takura T, Daida H. Cost-effectiveness of follow-up invasive coronary angiography after percutaneous coronary stenting: a real-world observational cohort study in Japan. BMJ Open. 2022;12:e061617. https://doi: 10.1136/bmjopen-2022-061617.
